# Hormonal and ultrasonographic characterization of the seasonal reproductive cycle of male and female *Crotalus durissus terrificus*

**DOI:** 10.21451/1984-3143-AR2017-0019

**Published:** 2018-12-05

**Authors:** Priscilla M. Matayoshi, Priscilla M. Souza, Vinícius P.O. Gasparotto, Michelle S. Araujo, Carla R.B. Simões, Fabiana F. Souza, Eunice Oba, Vânia M.V. Machado, Rui S.F. Júnior, Nereu C. Prestes

**Affiliations:** 1 Departamento de Reprodução Animal e Radiologia Veterinária, Faculdade de Medicina Veterinária e Zootecnia, Universidade Estadual Paulista “Júlio de Mesquita Filho”, UNESP, Botucatu, São Paulo, Brasil.; 2 Universidade Federal de Tocantins (UFT), Campus Araguaina, Araguaina, Tocantins, Brasil.; 3 Centro de Estudos de Venenos e Animais Peçonhentos (CEVAP), Universidade Estadual Paulista “Júlio de Mesquita Filho”, UNESP, Botucatu, São Paulo, Brasil.

**Keywords:** follicle, gestation, progesterone, rattlesnake, testosterone

## Abstract

Research concerning to characterize seasonal reproductive cycle in males and females of *Crotalus durissus terrificus* by ultrasound and hormonal measurement. Reproductive aspects (follicular and testicular cycles, and pregnancy) from 28 adult snakes (14 males and 14 females) during different months of the years were studied. Snakes housed individually in cages in an environment with controlled luminosity and humidity, and fed monthly. Females were examined by ultrasound during quiescence and active follicular phase, and pregnancy for follicular and embryo/fetal development. Males were evaluated to testicular echotexture and measurements during reproductive and non-reproductive season. The blood samples were collected from males and females for serum testosterone and progesterone determination, respectively. In 77% males the testes were identified by ultrasound and found increased size during summer, decreased serum testosterone in winter, and positive correlation between serum testosterone and testes size. There was no change in testicular echotexture in according to season. Testosterone concentration was decreased during winter and it was positively correlated with testes size. In 71% females, were observed follicular development (vitellogenesis) and gestation since winter to spring by ultrasonography. Parturition occurred mainly in summer. Pregnancy length was 123.0 ± 11.4 days, with mean 6.9 ± 1.5 newborns/female, and there was gradual increase of serum progesterone during this period. There was no variation in progesterone concentration in non-gravid females. Males and females Tropical Rattlesnake show seasonal variation of reproductive cycle and was clear a biennial cycle in female. The ultrasonography can be considered an essential tool to accomplish the follicular development, pregnancy and testicular alterations in Tropical Rattlesnake.

## Introduction

The ultrasound in reptile’s veterinary medicine is now a routine practice ( Grumbles and Rostal, 1997 ). It is considered a noninvasive technique, which provides valuable physiology of reproduction knowledge mainly when associated with blood hormonal analysis ( Lance *et al*., 2009 ), and was described in different species of snakes ( Almeida-Santos *et al*., 2004 ; Taylor and Denardo, 2005 ; Lind *et al*., 2010 ; Stahlschmidt *et al*., 2011 ; Banzato *et al*., 2013 ; Lind and Beaupre, 2014 ),

It is considered a noninvasive technique, which provides valuable physiology of reproduction knowledge mainly when associated with blood analysis ( Lance *et al*., 2009 ), and was described in different species of snakes ( Almeida-Santos *et al*., 2004 ; Taylor and Denardo, 2005 ; Lind *et al*., 2010 ; Stahlschmidt *et al*., 2011 ; Banzato *et al*., 2013 ; Lind and Beaupre, 2014 ), although there are few studies in *Crotalus durissus terrificus* (Almeida-Santos, 2004; Almeida-Santos, 2005 ). Descriptive studies concerning ultrasound monitoring and hormonal evaluation provide information regarding reproduction and the events during estrous cycle and pregnancy, being essential to captive snake handling. The captive reproduction of *Crotalus durissus terrificus* is an important practice once their venom is a natural source of bioactive substances with therapeutic potential, such as antitumoral properties, as well as for antiophidic serum production ( Cura *et al*., 2002 ; Soares *et al*., 2010 ; Calvete *et al*., 2011 ; Kumar *et al*., 2014 ; Nudel *et al*., 2012 ). The search for new drugs has indicated toxins from *Crotalus durissus terrificus* venom as inhibitors of cell adhesion, cell migration, epidermal tumor growth factor, metastases induced in experimental mice models, and use of some specific proteins for production of drugs ( Cura *et al*., 2002 ; Cunha *et al*., 2015 ; Neves *et al*., 2015 ; Reeks *et al*., 2015 ).

The better understanding of *Crotalus durissus terrificus* captive reproduction may contribute to the knowledge of their reproductive biology to improve handling conditions and to contribute with the production of compounds and new drugs obtained from its venom. Moreover, ultrasonography is a noninvasive diagnostic method, used to evaluate reproductive events as folliculogenesis, testicular measurement, pregnancy and sex determination ( Stetter, 2006 ; Augusto, 2007 ).

Thus, the main purposes of this study are to provide the reader about (1) the characterization of *Crotalus durissus terrificus* reproductive cycle using the ultrasonographic exam to follow seasonal changes of the reproductive structures and (2) correlate ultrasound images to serum levels of progesterone (pregnant and non-pregnant females) and testosterone (males) of captive rattlesnakes.

## Materials and Methods

This study was conducted in accordance with ethical guidelines recommended by National Council for Control of Animal Experimentation and College of Animal Experimentation, and it was approved by the Institution’s Animal Care and Experimentation Ethics Committee (Protocol Number 51/2010).Twenty-eight adult *Crotalus durissus terrificus* snakes (14 males and 14 females) with unknown age, were studied during April 2010 to April 2011 at the Center for the Study of Venoms and Venomous Animals (CEVAP). The snakes were rescued from wildlife and housed indoors, individually in polypropylene cages with water *ad libitum* . Rooms were acclimatized with temperature between 25°C and 27°C, 60-80% relative humidity and luminosity controlled in 9 hours of light and 15 hours of darkness. Food was offered monthly and consisted of one or two mice (*Mus musculus*).

Ultrasound exam and blood collection were made in different months of the year, according to reproductive seasonality of the species. Females were evaluated during follicular quiescence in April (fall), July (winter), October (spring) 2010 and January (summer) 2011; and during active follicular phase in April (fall), July (winter), September (spring), October (spring), December (summer) of 2010 and in January (summer) and April (fall) of 2011. During spring (September and October), females were evaluated two to three times, to follow follicular activity and subsequent pregnancy.

**Figure 1:**
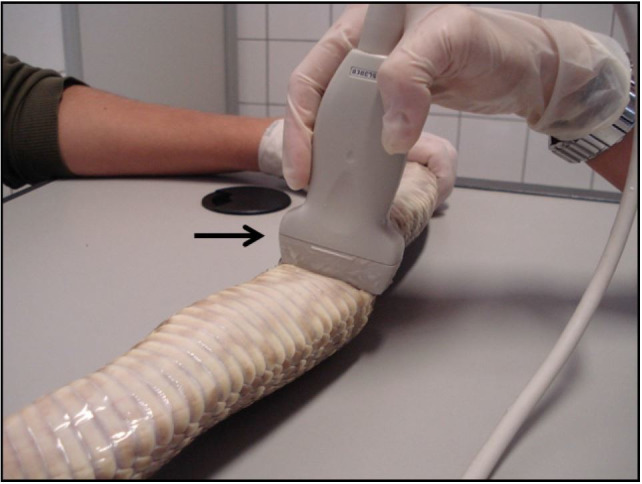
Transducer positioning to carry out ultrasound examination of snakes (*Crotalus durissus terrificus*) reproductive tract.

For blood collection and measurement of total length, snakes were handled with hook aid and restraint in a plastic tube. Blood samples were collected in the morning, by puncture of coccygeal vein, according to Lind *et al*. (2010) . After blood centrifugation, serum was separated and stored at -20°C until hormone measurement. Ultrasound exam was performed by physical restraint and on supine position, in B-mode (DP-3300Vet Mindray®, Mindray Medical International Limited, Baiwangxin, China) using a multifrequency linear transducer (7.5 to 10 MHz) positioned on the abdomen ( Fig. 1 ), according to Chiaraviglio *et al*. (1998) . Longitudinal and transverse planes images were obtained to evaluate testicular location, topography, shape, contour, size, echogenicity and echotexture, ovarian follicles and embryos/fetus development. Testes area, length and height were measured. The length and height were used to estimate testicular volume by a prolated spheroid formula [V = 4/3 π (1/2 x L) x (1/2 x H) ^2^] ( Ramirez-Bautista and Gutierrez-Mayen, 2003 ). Right and left testes volumes were polled in each month of the year.

Follicles were classified according to characteristics described at primary and secondary vitellogenesis ( Almeida-Santos, 2005 ; Bertona and Chiaraviglio, 2003 ; Betkowski, 2006 ; Yamanouye *et al*., 2004 ). In gravid females, follicles from pregnancy diagnosis to parturition were not evaluated. Embryo development was classified according to Pizzato (2006) and divided into three stages: 1) after ovulation, where only yolk is visible; 2) yolk abundant, where a small embryo is visible; and 3) embryo fully formed, without a visible yolk. Serum testosterone and progesterone concentrations were determined in males and females, respectively, by radioimmunoassay (COAT-A-COUNT, Siemens Medical Solutions Diagnostics, Los Angeles, CA, USA) according to manufacturer's recommendations.

Results were presented in mean ± standard error. T-test was used to compare all right and left testicles measurements in each season. There was no significant difference between right and left testicles, then the mean of each snake to compare the variable between seasons was used. One-way repeated measures ANOVA was used to analyze testosterone concentrations, body length, body weight, area, volume right/left length and height of testes during season; and Friedman repeated-measures analysis of variance on ranks to analyze body length and progesterone concentrations. Progesterone concentrations between gravid and non-gravid females were compared by Mann-Whitney test. All pairwise multiple comparisons were analyzed by Tukey’s test. Pearson correlation was used to correlate testosterone concentrations and right/left length, height, area and testes volume in males; and body length and number of offspring, progesterone concentrations and follicular size, fetal number counted by ultrasound and number of offspring in females. The correlation was considered weak (r = 0.10 to r = 0.30), moderate (r = 0.40 to r = 0.60) or strong (r ≥ 0.70). Significant differences were considered when P ≤ 0.05. Sigma-Stat 3.0 for Windows 2003 software was used for all statistical analyses.

## Results

**Figure 2:**
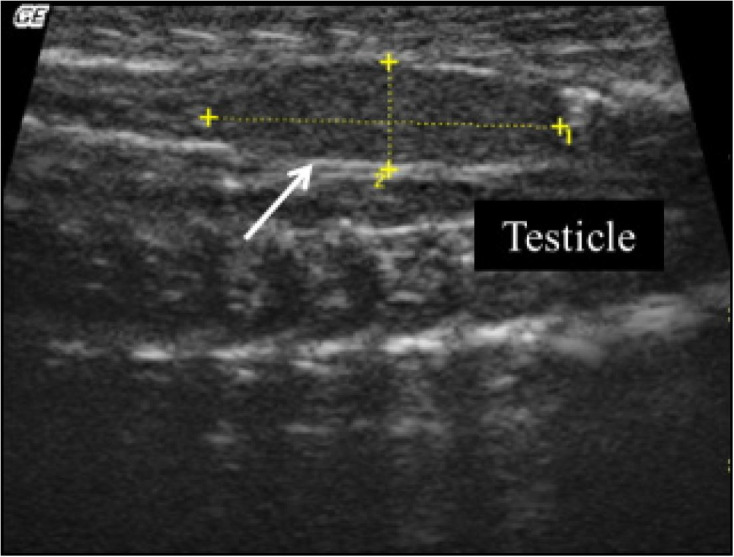
Left testis of a Tropical Rattlesnake (Crotalus durissus terrificus) in the last middle third of the coelom, cranial to kidney in a ventrolateral region to great vessels. Arrow indicates testis hyperechoic capsule.

Ultrasound exam allowed identifying both testes in 77% of males (n = 10 out of 14), which were positioned in ventrolateral region, near to great vessels ( Fig. 2 ), in middle third of the coelom, cranial to kidneys and arranged asymmetrically. Right testis was located more cranially than the left. Testes showed elongated or fusiform shape, regular and defined contour, homogeneous echotexture, hypoechoic in relation to adjacent structures and surrounded by a hyperechoic capsule. Testicular echotexture did not change during the seasons. The *vas deferens* was not examined.

**Figure 3:**
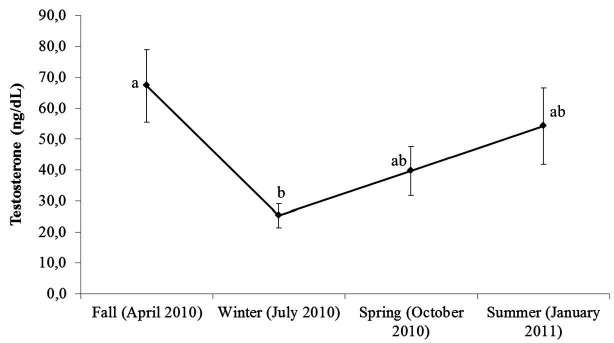
Testosterone concentrations of 14 snakes (*Crotalus durissus terrificus* ) during all seasons. Different letters indicate statistically significant differences at P < 0.05.

There was a seasonal variation in testes measurements with greater values during summer for length (ANOVA P = 0.001, F = 14.36), area (ANOVA P = 0.001, F = 7.33) and volume (ANOVA P = 0.001, F = 9.15) ( Table 1 ). However, height (ANOVA P = 0.008, F = 4.28) had a light reduction during winter then gradually increase until summer. Decreased testosterone concentrations were observed during winter ( Fig. 3 ).

**Table 1:** Mean ± standard error of length, height, area and volume of testes from 14 snakes (*Crotalus durissus terrificus*) during the seasons.

Measure/Season	Fall (April)	Winter (July)	Spring (October)	Summer (January)
Length (cm)	2.20 ± 0.07^a^	2.14 ± 0.09^a^	2.09 ± 0.08^a^	2.76 ± 0.09^b^
Height (cm)	0.61 ± 0.02^ab^	0.57 ± 0.03^a^	0.60 ± 0.04^b^	0.66 ± 0.03^a^
Area (cm^2^)	1.36 ± 0.08^a^	1.27 ± 0.10^a^	1.25 ± 0.09^a^	1.81 ± 0.12^b^
Volume (cm^3^)	0.45 ± 0.04^ab^	0.35 ± 0.04^a^	0.31 ± 0.03^a^	0.59 ± 0.07^b^

Different letters in the same line indicate statistical difference at P ≤ 0.05.

Positive correlation was found between testosterone concentration and testes size during fall (right testis: length r = 0.62, P = 0.017, height r = 0.71, P = 0.005, volume r = 0.56, P = 0.08), winter (right testis: length r = 0.63, P = 0.017) and summer (right testis: area r = 0.60, P = 0.041, volume r = 0.60, P = 0.025; left testis: area r = 0.80, P < 0.001, length r = 0.56, P = 0.047, height r = 0.77, P = 0.004, volume r = 0.80, P < 0.001).

In males, body weight mean ± SE was 805.0 ± 72.1 g (ranging from 500.0 to 1,300.0 g) and length 108.5 ± 3.7 cm (ranging from 89.0 to 131.0 cm).

**Figure 4:**
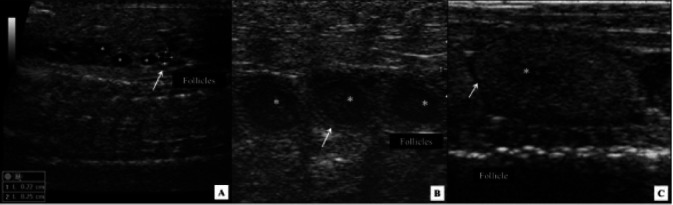
Ultrasound image of a snake (*Crotalus durissus terrificus*) ovary. A. Non-vitellogenic follicles. B. Circular or oval shaped vitellogenic follicles (*) of 10.0 mm in diameter arranged in chain observed during fall season. Note follicles bordered by a hyperechoic capsule (arrow). C. Vitellogenic follicles (*) with oval shape (15 x 26 mm) and lined with a moderate quantity of liquid.

In females, circular or oval shaped ovaries were found, with follicles arranged in chain, located in the middle region of coelomic cavity, caudally to the gall bladder and interspersed by adipose tissue ( Fig. 4A ). Ovarian morphology was similar in all females during the period. During the first evaluation (fall) 10.0 mm in diameter follicles (vitellogenic or in primary vitellogenesis) were observed, which showed circular or oval shaped, with hyperechoic borders and hypoechoic center, and homogeneous or slightly heterogeneous echotexture. During winter (July) increased follicles diameter (18.0 to 26.0 mm) were observed and females were considered to be in secondary vitellogenesis (n = 10 gravid females out of 14 total). Follicles showed a circular to oval shape, arranged in a chain or grouped in cluster ( Fig. 4B ), with echotexture similar to that observed in fall. The follicles were lined with moderated amounts of liquid ( Fig. 4C ).

**Figure 5:**
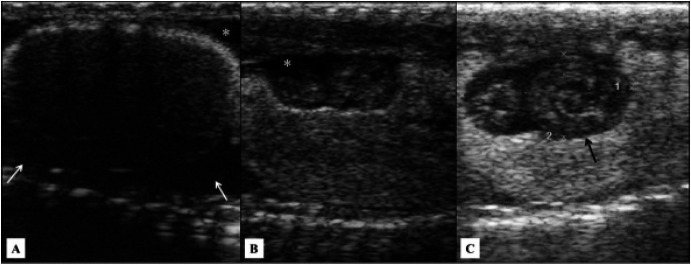
Ultrasound image of a snake (*Crotalus durissus terrificus*) oviduct containing zygote and embryo. A. Zygote characterized as flattened, hypoechoic and oblong structures with homogeneous echotexture (arrows) lined with fluid (*). B. Note the same structure with a peripheral mass partially filled with liquid (*), representing the first stage of embryonic development. C. Embryo (arrow) in second stage of embryonic development.

**Figure 6:**
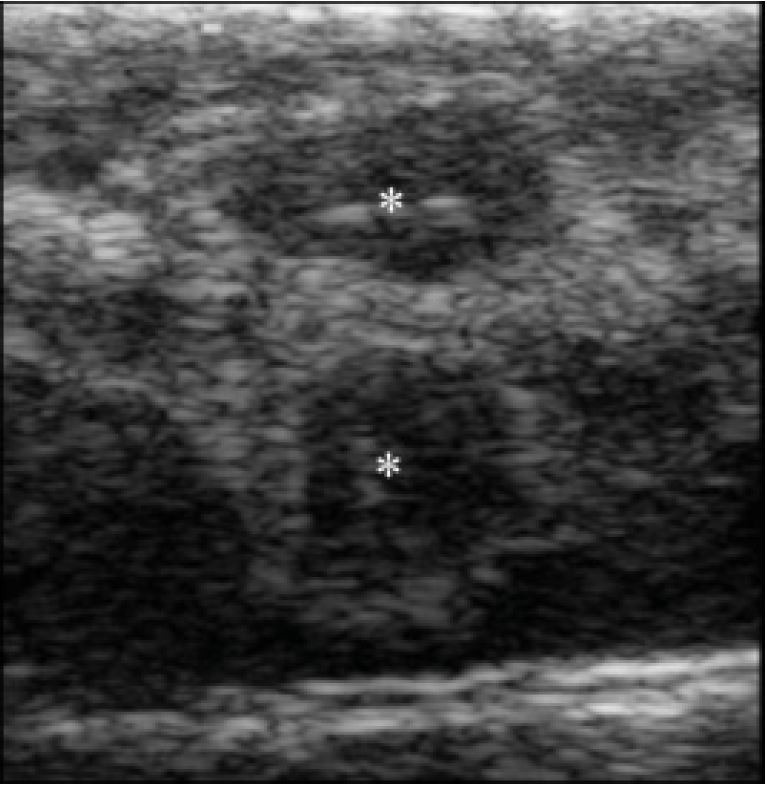
Ultrasound of snake (*Crotalus durissus terrificus*) ovary showing atresic follicles (*).

During spring (September) marked follicular development in approximately 71% females (n = 10 out of 14 total) was observed. Atresic follicles were observed in some females. Ovaries contained also hypoechoic structures, homogeneous echotexture and characteristics of pre-ovulatory follicles, measuring approximately 30 mm. Oblong, hypoechoic and flattened structures were also found, with homogeneous echotexture, lined with liquid and larger than 30 mm (30-40mm) ( Fig. 5A ). These females were assessed more frequently and we noted fluid inside of these structures, which were classified as zygote. Followed 1–2 weeks, embryos were identified on vesicles periphery. Embryo development was monitored and all stages were observed ( Fig. 5B - 5C ). Stages 1 and 2 were observed during spring (September). The onset of bones calcification, especially the skull, and heartbeats were visualized during the following month (October, spring). The amount of vitellus was reducing gradually with advancing gestation. In other females, were found hypoechoic flattened structures, with irregular surface and hyperechoic borders, which formed posterior acoustic shadow that were classified as follicles in regression (atresia) ( Fig. 6 ).

**Figure 7:**
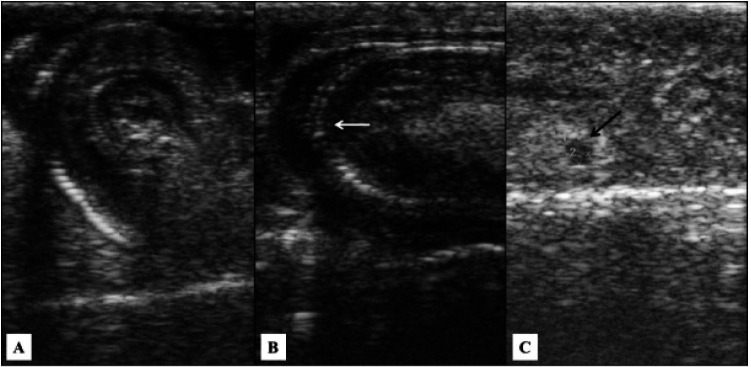
Ultrasound of a snake (*Crotalus durissus terrificus*) oviduct and ovary showing pronounced fetal development with spine calcification or absence of vitellus (A and B). C. Follicle circular and anechoic in postpartum period (arrow).

In December and January (summer) the females were in different stages of gestation, in which we observed calcification of fetal spine ( Fig. 7A - 7B ), forming a posterior acoustic shadow. In this period, we noted also the absence of vitellus and discrete fetal movements. During this period, fetal count was not possible. In two females, parturition occurred in December (summer). Parturition of most gravid females occurred in January (summer). After parturition, ovaries contained circulars, anechoic and arranged in chain pre-vitellogenic follicles with size less than 10 mm ( Fig. 7C ). In April (fall of 2011) all females had small anechoic and pre-vitellogenic follicles.

Four females were non-gravid, of which two had low levels of serum progesterone, few, circular and anechoic small follicles during all months of evaluation. Remaining females started secondary vitellogenesis, but ovulation was not observed. There was follicular growth in April (fall) and July (winter), with follicles reaching pre-ovulatory size but then regressed from the assessment of September (spring). In January (summer), follicles were small, circular and anechoic.

Positive pregnancy diagnosis was based on elevated serum progesterone concentrations which was confirmed by ultrasound exam. Mean length of pregnancy was 123.0 ± 11.4 days (range 96-137 days), with birth of 69 newborns, mean 6.9 ± 1.5 newborns/female (1-14 newborns). Fetal number was determined by ultrasound exam in all females, except one, which had the greatest number of fetuses. A correlation between litter size and body length was not find (r = 0.51, P = 0.13). However, there was strong correlation (r = 0.98, P < 0.0001) between fetuses counted by ultrasound and number of newborns.

**Figure 8:**
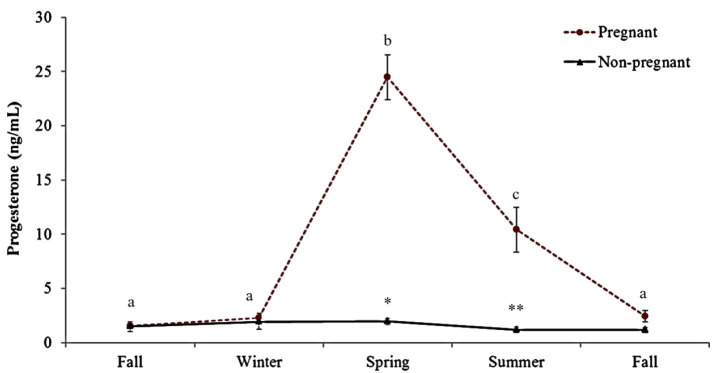
Progesterone concentrations of 10 gravid and 4 non-gravid female snakes (*Crotalus durissus terrificus*) during reproductive and non-reproductive season. Different letters indicate significant differences at P < 0.05 in the group. (*) indicate P = 0.007 between groups in spring; (**) indicate P = 0.004.

To analysis progesterone concentrations, two values were removed in gravid (155.2 ng/mL in the spring) and non-gravid females (123.82 ng/mL in spring) for being outliers. Serum progesterone concentrations varied according to season (Friedman, P < 0.001) and significantly between gravid and non-gravid females ( Fig. 8 ). Progesterone levels during ovulation were 15.2 ng/mL. A moderate positive correlation (r = 0.40, P < 0.0001) was observed between follicular size and progesterone concentrations. Mean snout-vent length ± standard error of females was 88.0 ± 3.3 cm (range 61-99 cm).

## Discussion

Chemical restraint can be indicated for aggressive or very active snakes for ultrasound exams ( Betkowski, 2006 ; Chiaraviglio *et al*., 1998 ; Gnudi *et al*., 2009 ), though snake retention only with a plastic tube in our study was efficient for testicles visualization by ultrasound. Testicular topography observed is consistent with reported in another studies ( Chiaraviglio *et al*., 1998 ; Neto *et al*., 2009 ; Redrobe and Wilkinson, 2002 ). Despite this, one testis in some males was not found, which may have occurred due to air accumulation between scales during ecdysis and the presence of gas and feces in intestine ( Isaza *et al*., 1993 ; Raiti, 2000 ), producing reverberation artifacts. In some snakes, fat in coelomic cavity hindered testes location because of the similar echogenicity, as described in other species ( Isaza *et al*., 1993 ; Neto *et al*., 2009 ; Raiti, 2000 ).

The morphology and testicular echotexture found were similar to those described in literature ( Chiaraviglio *et al*., 1998 ; Neto *et al*., 2009 ; Redrobe and Wilkinson, 2002 ). Echotexture testes did not suffer seasonal influence, but greater sizes were found closer to the breeding season (summer). These findings were consistent with increased spermatogenic activity in Tropical Rattlesnakes that occurs between September to March, being higher in January in the southern hemisphere, and testicular regression in winter ( Almeida-Santos, 2005 ; Salomão and Almeida-Santos, 2002 ). Increased testicular activity was also related to pregnancies, which occurred in October. Despite the ease to locating testes, *vas deferens* were not able to be observed, which is often possible only in larger snakes ( Redrobe and Wilkinson, 2002 ).

As testicular size, testosterone concentrations were affected by seasonal influence, with lower values of testes measurements observed during the winter and spring, and higher values in summer, which corroborated with other studies ( Almeida-Santos *et al*., 2004 ; Zacariotti, 2004 ). In other species of same genus, seasonality was also observed, but higher testosterone concentrations are described in fall and spring ( Hoss *et al*., 2011 ). Although the elevation of testosterone levels did not coincide with testes size increase, a gradual hormone increase was observed since winter to summer, with a peak during fall. Already testes size was higher in the summer, though the testicular volume remained higher during fall, similar to testosterone concentrations. Furthermore, viper’s reproduction events not always match, when we consider males and females and events related to the development of vitellogenesis, spermatogenesis, breeding, production and hormone secretion ( Schuett, 1992 ). There is a higher asynchrony in these events ( Schuett, 1992 ). Nevertheless, testosterone levels were positively correlated to testicular dimensions, especially during summer and fall, which can be a difference between *in-situ* and captive animals.

Ovaries were located easily in all females, although some authors reported greater difficulty in snakes during follicular quiescence ( Redrobe and Wilkinson, 2002 ). Seasonal female’s reproductive cycles, with an active phase of follicular growth, mating, pregnancy, parturition in a year followed by follicular quiescence in the next year, confirmed the two-year cycle already described by other authors for Tropical Rattlesnakes ( Almeida-Santos and Orsi, 2002 ). In females that became gravid, primary vitellogenesis ( Almeida-Santos, 2005 ; Bertona and Chiaraviglio, 2003 ; Betkowski, 2006 ) was observed during mid-fall, which corresponded to lower progesterone concentrations (1.57 ± 0.17 ng/mL) and small follicles (11.64 ± 0.47 mm). These concentrations were similar to those females who did not become gravid (1.47 ± 0.45 ng/mL), however follicle diameters were lower (5.70 ± 1.70mm).

Follicular development was observed during secondary vitellogenesis (winter), especially in females that became pregnant; although mean of progesterone concentration was similar between gravid (2:29 ± 0:43 ng / ml) and non-gravid (1.90 ± 0.66 ng/mL). These results corroborated with Almeida-Santos (2005) , which also featured the secondary vitellogenesis, however, we expected a rise in progesterone levels ( Almeida-Santos, 2005 ) due to growth in oviductal epithelium ( Girling, 2002 ).

During spring (September) there was marked increase in diameter and follicular echotexture, suggestive of pre-ovulatory vitellogenesis ( Yamanouye *et al*., 2004 ). At this stage, counting follicles to determine litter size can lead to an erroneous value and overestimation, since some follicles can become atretic ( Taylor and Denardo, 2005 ). During this period, there was a peak in serum progesterone concentrations in gravid females, which was expected, since ovulation occurs during this phase. Although there is controlled handling in captivity, such as food availability, it appears that ovulation occurs in the same period that specimens living *in-situ* ( Almeida-Santos and Salomão, 1997 ).

According to Denardo and Autumn (2001) , after ovulation it is possible to observe aligned, circular or oblong mass greater than 30 mm, occupying most distal part of the coelomic cavity. In our study, the largest diameter recorded was 44 mm. Probably, this mass corresponds to an oocyte or an egg (zygote) in the oviduct, as described by Betkowski (2006) . During this period, we found also elevation of progesterone concentration, only in gravid females, indicating ovulatory phase ( Almeida-Santos, 2005 ).

We expected that larger testicular size and increased testosterone production precede and remain during ovulation period. Although we have not followed sperm production, we believe that a greater number of sperm cells coinciding with the increase in testicular volume. In fact, the hypothesized spermatogenesis in genus *Bothrops* has been described during summer and spring, and courtship and mating in the fall ( Almeida-Santos and Salomão, 2002 ), presumably because many species of snakes, including *Crotalus durissus terrificus* are able to store sperm in female reproductive tract for several months for prolonged period, prior to ovulation during winter ( Almeida-Santos and Salomão, 1997 ), which justifies our findings. Moreover, testosterone peak was observed in fall and can induce to social interaction, male-male fighting and pre-copulatory guarding, which can precede copulation (reviewed by Almeida-Santos and Salomão, 2002 ). These social behaviors have been described in *Crotalus durissus terrificus* from South America during fall and early winter ( Almeida-Santos and Salomão, 1997 ; Almeida-Santos *et al*., 1998 ), which coincide with testosterone peak observed in snakes, and in *ex-situ* snakes from the same species ( Almeida-Santos and Salomão, 1997 ).

During the summer (December and January), most of females were in late pregnancy. In fact, gestation is described during spring and summer (from October to March in South hemisphere) and parturition in summer in studies conducted with females captured from natural habitat ( Almeida-Santos and Salomão, 2002 ), as our results observed in captivity conditions.

Fetal movement and absence of vitellus were the main findings to indicate gestational phase and peripartum period ( Taylor and Denardo, 2005 ). Progesterone concentrations remained high during this period only in gravid females, which is characteristic of this species during pregnancy ( Almeida-Santos *et al*., 2004 ).

Highest progesterone in gravid *vs*. non-gravid females indicate that this hormone’s concentrations can be used for gestational diagnosis in this species (Fig. 9). As in mammals, serum progesterone remains high during all stages of pregnancy in reptiles and is responsible for maintaining gestation ( Almeida-Santos *et al*., 2004 ). However, studies with viviparous snakes have shown that exogenous administration of this hormone does not influence the duration of pregnancy, suggesting that the fetus is responsible for parturition stimulus ( Bonnet *et al*., 2001 ), irrespective of progesterone concentrations. Progesterone levels decrease drastically in postpartum of snakes ( Almeida-Santos, 2005 ), a fact confirmed in our study.

Almeida-Santos and Orsi (2002) described that females postpartum enter a quiescent phase (primary vitellogenesis) and will be in active stage (secondary vitellogenesis) only at the next season, featuring a biennial cycle. This period during the cycle is apparently related to ability to store fat in the coelomic cavity, aiming at future reproduction ( Blem, 1982 ). In first and second fall period, progesterone concentrations were baseline values in gravid and non-gravid females, showing a new cycle and primary vitellogenesis ( Almeida-Santos, 2005 ).

Number of newborns was similar to the mean known for vipers (2-16 pups/female) ( Seigel and Ford, 1987 ). A significant correlation between litter size and female body size has been documented in a wide variety of reptiles (e.g., Shine, 2003 ; January-Cinquini, 2004 ; Almeida-Santos, 2005 ), but in our studies, there was no correlation. Pizzatto (2006) also did not find a correlation between number of vitellogenic follicles or embryos and female size in *Epicrates species cenchria assisi*, *Epicrates c. cenchria* and *Epicrates c. crassus*. Ultrasound exam is not considered a fully safe technique to quantify the number of fetuses in some species ( Root and Spaulding, 1994 ), but we found strong correlation between fetal count by ultrasonography and the number of newborns, suggesting that in Tropical Rattlesnakes this method is reliable and secure, except in snakes with large fetal number.

It cannot be affirmed that the four females which did not become pregnant during this study were gravid in the year before, since they were wild animals. In these females, folliculogenesis was observed, although ovulation did not occur. Also, there are no elevation in serum progesterone, which confirms the hypothesis of an anovulatory cycle and follicular quiescence stage ( Almeida-Santos *et al*., 2004 ). Sexual immaturity could also explain follicular quiescence, but these females had the same body size that gravid animals did, although sexual maturity is related to body reserves ( Bonnet *et al*., 2001 ). Stress is another factor that may have interfered in vitellogenesis for these four females, which can change their reproductive behavior ( Greenberg and Wingfield, 1987 ; Shine, 2003 ).

## Conclusion

In conclusion, in this study we observed seasonal anatomical changes in the reproductive structures of male and female Tropical Rattlesnakes by ultrasound exam and serum testosterone and progesterone variation. Ultrasound exam was efficient to identify reproductive structures, such as pre-vitellogenic and vitellogenic follicles, embryos, follicular atresia and testes, and diagnosis and monitoring of pregnancy and can be a useful tool in the management of snakes. Furthermore, progesterone concentration can be used to diagnose gestation in this snake species.
